# Predicting patient-reported outcomes following hip and knee replacement surgery using supervised machine learning

**DOI:** 10.1186/s12911-018-0731-6

**Published:** 2019-01-08

**Authors:** Manuel Huber, Christoph Kurz, Reiner Leidl

**Affiliations:** 1 German Research Center for Environmental Health, Institute for Health Economics and Health Care Management, Helmholtz Zentrum München, Postfach 1129, 85758 Neuherberg, Germany; 2 Munich Center of Health Sciences, Ludwig-Maximilians-University, Ludwigstr. 28, 80539 Munich, RG Germany

**Keywords:** Patient-reported outcomes, Hip replacement, Knee replacement, Shared decision-making, Machine learning, Binary classification, Predictive performance, Variable importance, Boosting

## Abstract

**Background:**

Machine-learning classifiers mostly offer good predictive performance and are increasingly used to support shared decision-making in clinical practice. Focusing on performance and practicability, this study evaluates prediction of patient-reported outcomes (PROs) by eight supervised classifiers including a linear model, following hip and knee replacement surgery.

**Methods:**

NHS PRO data (130,945 observations) from April 2015 to April 2017 were used to train and test eight classifiers to predict binary postoperative improvement based on minimal important differences. Area under the receiver operating characteristic, J-statistic and several other metrics were calculated. The dependent outcomes were generic and disease-specific improvement based on the EQ-5D-3L visual analogue scale (VAS) as well as the Oxford Hip and Knee Score (Q score).

**Results:**

The area under the receiver operating characteristic of the best training models was around 0.87 (VAS) and 0.78 (Q score) for hip replacement, while it was around 0.86 (VAS) and 0.70 (Q score) for knee replacement surgery. Extreme gradient boosting, random forests, multistep elastic net and linear model provided the highest overall J-statistics. Based on variable importance, the most important predictors for post-operative outcomes were preoperative VAS, Q score and single Q score dimensions. Sensitivity analysis for hip replacement VAS evaluated the influence of minimal important difference, patient selection criteria as well as additional data years. Together with a small benchmark of the NHS prediction model, robustness of our results was confirmed.

**Conclusions:**

Supervised machine-learning implementations, like extreme gradient boosting, can provide better performance than linear models and should be considered, when high predictive performance is needed. Preoperative VAS, Q score and specific dimensions like limping are the most important predictors for postoperative hip and knee PROMs.

**Electronic supplementary material:**

The online version of this article (10.1186/s12911-018-0731-6) contains supplementary material, which is available to authorized users.

## Background

Shared decision making (SDM) is an approach where clinicians and patients share available evidence and preferences to support upcoming treatment decisions [1]. SDM has been found to improve care and reduce costs [2]. A recent Cochrane review for the effects of decision aids included 105 studies (31,043 patients in total) and concluded that while knowledge perception increased, no adverse effects on outcomes or satisfaction were observed [3]. One way to support SDM is to gather and evaluate patient reported outcome measures (PROMs). These are powerful tools which transform symptoms into numerical scores that capture why most patients seek medical attention, namely to improve their health state [4]. To control quality of care the National Health Service (NHS) routinely collects PROMs for four elective procedures since 2009 [5] and a the majority of Swedish quality registers are obliged to gather PROMs as well [6]. One advantage of individual PROMs compared with average study population results, is the possibility to predict individual outcomes [7]. While prediction models exist for reoperations [8], scheduling [9, 10] or morbidity risk [11, 12] of elective surgery, models that predict health-related quality of life are rare, despite around 160,000 hip and knee replacement procedures that are conducted in England and Wales every year [13]. To support SDM, accurate prediction models are needed, for example to inform doctors and patient about likely surgery outcomes. While generalized linear models are solid tools, machine-learning techniques are often able to outperform linear approaches [14–17]. Combining machine learning with expertise from clinicians is needed to improve collective care and to foster precision medicine [18]. However, there is no free lunch in optimization [19, 20] and thus, no single model works best for all problems. Moreover, machine-learning models are often seen as black boxes that deliver very good performance but are less intuitive and transparent than traditional statistical methods. Additional uncertainty is partly rooted in the nature of machine learning where modelers have a wide variety of algorithms and approaches to choose from [21], unless more automated approaches are implemented [22]. Gaining and sharing empirical experience is therefore key to advance the understanding of model applicability and usefulness in respective scenarios. Despite thousands of papers for machine learning in medicine, meaningful contribution to clinical care is still rare [23]. The aim of this study is to evaluate eight different machine learning and one generalized linear model to predict binary PROM outcome following hip and knee replacement surgery. Moreover, by evaluating variable importance of respective models, we provide easy-to-interpret evidence illustrating model findings.

## Methods

### Data

The NHS publishes PROMs data for hip replacement, knee replacement, varicose vein and groin hernia on a monthly basis and releases a finalized data set every year [24]. Eligible patients are only those who are treated by or on behalf of the NHS. The PROMs program is mainly limited to England. NHS PROMs data from April 1st 2015 to March 31st 2017 were used to train and test models. The data sets contain 81 variables before filtering. Variables include sociodemographics with living status, age groups, disease affliction by self-report (“Have you been told by a doctor that you have …? ”), EQ-5D-3L [25], visual analog scale (VAS), Oxford Hip Score (OHS) [26] dimensions, Oxford Knee Score (OKS) [27] dimensions and respective Q scores (sum of OHS or OKS). We removed observations with missing values or variables with near zero or zero variance. Moreover, we removed all post-operative variables except those of interest (VAS and Q score). Plausibility checks were applied to all variables. Some algorithms are sensitive to data imbalances. Three common options exist to address this issue, downsampling, upsampling and Synthetic Minority Over-sampling Technique (SMOTE) [28]. Downsampling removes observations from the majority class, upsampling randomly increases observations from the minority class and SMOTE is a more complex form of oversampling that artificially creates minority cases using nearest neighbors. We disregarded downsampling because it causes loss of information. One disadvantage of SMOTE is that it can add additional noise to the dataset because of increased overlap between classes. Due to its ease of use and high competitiveness [29] compared with more complex techniques, we chose normal upsampling to reach balanced class ratios. Normal upsampling is associated with two disadvantages. One, it makes overfitting more likely since it replicates the minority class. Two, it increases the number of observations and thereby increases training time. To avoid overfitting we use cross-validation and apply upsampling only to the training but not to the test data. The increase of computational time was acceptable for us.

### Model selection, outcome metrics, cross-validation and variable importance

Algorithm selection has significant influence on model outcome and is essential for model performance [30]. Due to the vast amount of available algorithms – the caret package [31] in R currently (May 2018) includes 237 models of which 189 can be used for classification problems – it is difficult for researchers to know in advance which algorithm performs best. To reduce the number of potential test algorithms, several software environments offer so called cheat sheets that provide some guidance on algorithm implementation for specific problems [32–34]. These cheat sheets are mainly based on expert experience but also oversimplification and generalization. Moreover, data cleaning, feature engineering, hyper-parameter tuning and ensembling cause additional complexity. To select models, we also incorporated expertise published in supplement 1 of Sauer et al. 2018 [35]. The following algorithms were selected for comparison: logistic regression, extreme gradient boosting [36], multi-step adaptive elastic-net [37], random forest [38], neural net [39, 40], Naïve Bayes [41], k-Nearest Neighbors [42] and boosted logistic regression [43]. Carets pre-defined grid search values for respective algorithm hyper-parameters were used. Originally, a support vector machine with radial basis function kernel [44] has been evaluated as well. However, due to functional instabilities, results were inconsistent and we consequentially removed the implementation from the analysis.

The area under the receiver operating characteristic (AUROC) is used as outcome metric for the training set. For binary classification, the AUROC combines the sensitivity, in our case the probability of correctly classifying a patient who will reach the minimal important difference (MID), and its specificity, i.e. the probability of correctly classifying a case that will stay below MID. The AUROC combines both characteristics at different probability cutoff points. It has certain advantages compared with overall accuracy, e.g. it is not dependent on decision thresholds or prior class probabilities [45]. It ranges from 0.5 (random predictor) to 1 (perfect predictor). To validate our models and to detect possible overfitting, we test the classifiers with surgery outcomes of the 2016/2017 full data release for both procedures. Since neither cost nor utility nor loss functions for the test characteristics (confusion matrix) are available, we value sensitivity (true positives / (true positives + false negatives); the proportion of people correctly predicted to have improvement among all patients who have improvement) and specificity (true negatives / (true negatives + false positives); the proportion of people correctly predicted to have no improvement among all patients who have no improvement) the same. We also provide the Youden J-statistic [46] (Sensitivity + Specificity – 1) for each training model. The statistic is calculated across different thresholds (0 to 1 by steps of 0.05) and allows selecting the threshold that maximizes the sum of sensitivity and specificity. It ranges from − 1 to + 1 and a higher score is considered better. For the validation models we also report other common metrics like positive predictive value/precision (the proportion of patients correctly predicted to have improvement compared with all patients predicted to have improvement), negative predictive value (the proportion of patients who are correctly predicted to have no improvement compared with all patients predicted to have no improvement), F1-score (2 * (Recall * Precision) / (Recall + Precision); a balanced average of precision and sensitivity) and balanced accuracy (0.5 * (true positives / N positives + true negatives / N negatives); the average proportion of correctly classified cases across patients with actual improvement and no improvement).

Overfitted models predict outcomes based on spurious correlations or random noise and have poor fit with unseen data. To avoid overfitting, we used five-fold repeated cross-validation (CV). For five-fold CV, data are split into five equally big parts. One part is retained and the other four parts are used for training. Once training is finished, model performance is tested with the retained part. This is iterated until each of the parts has been used for validation once. Seeds were set to make results reproducible and models comparable.

Variable importance is a concept to indicate the importance of each variable for the predictive performance of the model. For example, in the case of extreme gradient boosting, the importance is calculated by permuting each predictor variable and summing the importance (change in accuracy) over each boosting iteration [47]. The scaled importance ranges from 0 (unimportant variable) to 100 (most important variable). We calculate variable importance for models where the function is available, namely extreme gradient boosting, multistep elastic net, random forest, neural net and linear model.

## Performance comparison

For validation and comparison purposes we benchmark one of our high performing hip models against the hip prediction model used by the NHS (predictions of the NHS model are included in the released dataset). The NHS model [48] is a linear regression model that has access to more detailed variables (e.g. age instead of age groups). Since it predicts actual postoperative outcome values, we use two different approaches to benchmark performance. First, we transform the absolute NHS predictions into binary form, by evaluating if the predicted postoperative value reaches MID (= improvement) or not (= no improvement). Second, we calculate our own regression model based on the respective implementation used for the first comparison, via 10-fold cross validation (3 repetitions) and we compare it against the regression results of the NHS model. Comparison metrics for the regression models are root mean squared error (RMSE) and mean absolute error (MAE).

### PROMs

The NHS uses the EQ-5D-3L [25] including its VAS, the OHS [26] and the OKS [27] to collect PROMs for hip and knee replacement surgery. The EQ-5D-3L is a widely accepted and validated instrument to measure HRQoL. It consists of five questions, also called dimensions, and the VAS. The five dimensions include mobility, self-care, usual activities, pain/discomfort and anxiety/depression. The survey taker has three answer possibilities (no problems, some/moderate problems, unable to or extreme problems). Moreover, the survey taker is asked to mark his current health state on the VAS. The VAS ranges from 0 (worst imaginable health state) to 100 (best imaginable health state). The VAS measures a broader construct of health and is closer to the patient perspective than population based value sets that are normally used to transform health states. Oxford Hip Score (OHS) as well as Oxford Knee Score (OKS) are hip and knee specific instruments to measure disease-specific HRQoL. They consist of 12 questions with five answer possibilities. Values from 0 (severe) to 4 (none) are assigned to each answer and get summed up to the Q score. The sum score grades are 0–19, 20–29, 30–39 and 40–48 points and can be translated to severe/moderate/mild-to-moderate arthritis and satisfactory joint function. Patients complete the preoperative survey in the interval between having an appointment/being fit for surgery and the procedure. The time lag between pre- and postoperative questionnaires is at least 6 months. The surveys are voluntary and the response rate is around 75%.

### Minimal important differences (MIDs)

MIDs describe the change of a measure that is detectable by the patient. MIDs are not universally valid and vary by patient group and instrument [49]. Several ways to calculate MIDs for PROMs exist. They include anchor-based methods, clinical-trial-based methods as well as distribution-based methods [50]. 0.5 standard deviations were found to approximate MIDs for HRQoL in chronic diseases very well [51]. Since we had no clinical data, we used half a standard deviation of baseline preoperative VAS as MID. This resulted in VAS MIDs of 11 (hip) and 10 (knee). Using multiple anchor-based approaches, a study from Denmark calculated hip MIDs that ranged from 5 to 23 [52]. Our MID is within this range. The individual MIDs for OHS and OKS were taken from literature, they were 8 and 7 respectively [53].

## Results

Table 1 depicts sociodemographic data and patient perception before and after surgery. In total, 30,524 observations for hip and 34,110 observations for knee replacement surgery were included from the training dataset 2015/2016. 59.7 and 56.44% of patients were female, respectively. Over 70% of hip and knee surgery patients were between 60 and 79 years of age. Around 7 to 8% had related surgery before. The majority of both patient groups considered themselves to have a disability. On average, patients before hip replacement had lower generic (64.85) and disease specific (18.47) health perception compared with patients before knee replacement (68.18; 19.34) but average postoperative outcomes were higher for hip patients. The numbers for the testing dataset 2016/2017 are comparable. Only slightly more surgeries were done in 2016/2017 and the percentage of people with VAS improvement increased by around 2 percentage points.

**Table 1:** Demographics and health perception of hip and knee patients

Hip replacement surgery	2015/2016	2016/2017
Observations	30,524	31,905
Female	18,224 (59.7%)	19,009 (59.58%)
Age band (years)		
20 to 29	5 (0.02%)	0
30 to 39	22 (0.07%)	0
40 to 49	576 (1.89%)	457 (1.43%)
50 to 59	3819 (12.51%)	4204 (13.18%)
60 to 69	10,633 (34.83%)	10,898 (34.16%)
70 to 79	11,607 (38.03%)	12,179 (38.17%)
80 to 89	3844 (12.59%)	4130 (12.94%)
90 to 120	18 (0.06%)	37 (0.12%)
Previous hip-replacement surgery	2481 (8.13%)	1587 (4.97%)
Disability	16,654 (54.56%)	16,899 (52.97%)
Mean preoperative VAS score	64.85 (±21.94)	64.35 (±22.30)
Mean postoperative VAS score	76.91 (±18.17)	77.61 (±17.66)
Mean preoperative Q score	18.47 (±8.34)	18.19 (±8.31)
Mean postoperative Q score	39.66 (±8.62)	39.74 (±8.65)
Patients with improvement (VAS)	13,321 (43.64%)	14,512 (45.49%)
Patients with improvement (Q score)	27,636 (90.54%)	29,026 (90.98%)
Knee replacement surgery	2015/2016	2016/2017
Observations	34,110	34,406
Female	19,253 (56.44%)	19,483 (56.63%)
Age band (years)		
40 to 49	43 (0.13%)	19 (0.05%)
50 to 59	3368 (9.87%)	3552 (10.32%)
60 to 69	13,025 (38.19%)	12,716 (36.96%)
70 to 79	13,849 (40.60%)	13,974 (40.62%)
80 to 89	3825 (11.21%)	4145 (12.05%)
90 to 120	0	0
Previous knee-replacement surgery	2348 (6.88%)	1194 (3.47%)
Disability	17,964 (52.66%)	17,576 (51.08%)
Mean preoperative VAS score	68.18 (±20.24)	67.86 (±20.36)
Mean postoperative VAS score	74.27 (±18.61)	74.81 (±18.40)
Mean preoperative Q score	19.34 (±7.79)	19.32 (±7.71)
Mean postoperative Q score	35.60 (±9.51)	35.83 (±9.37)
Patients with improvement (VAS)	11,037 (32.08%)	11,679 (33.94%)
Patients with improvement (Q score)	28,657 (84.01%)	29,096 (84.57%)

Percentage or standard deviation in brackets

**Fig. 1:**
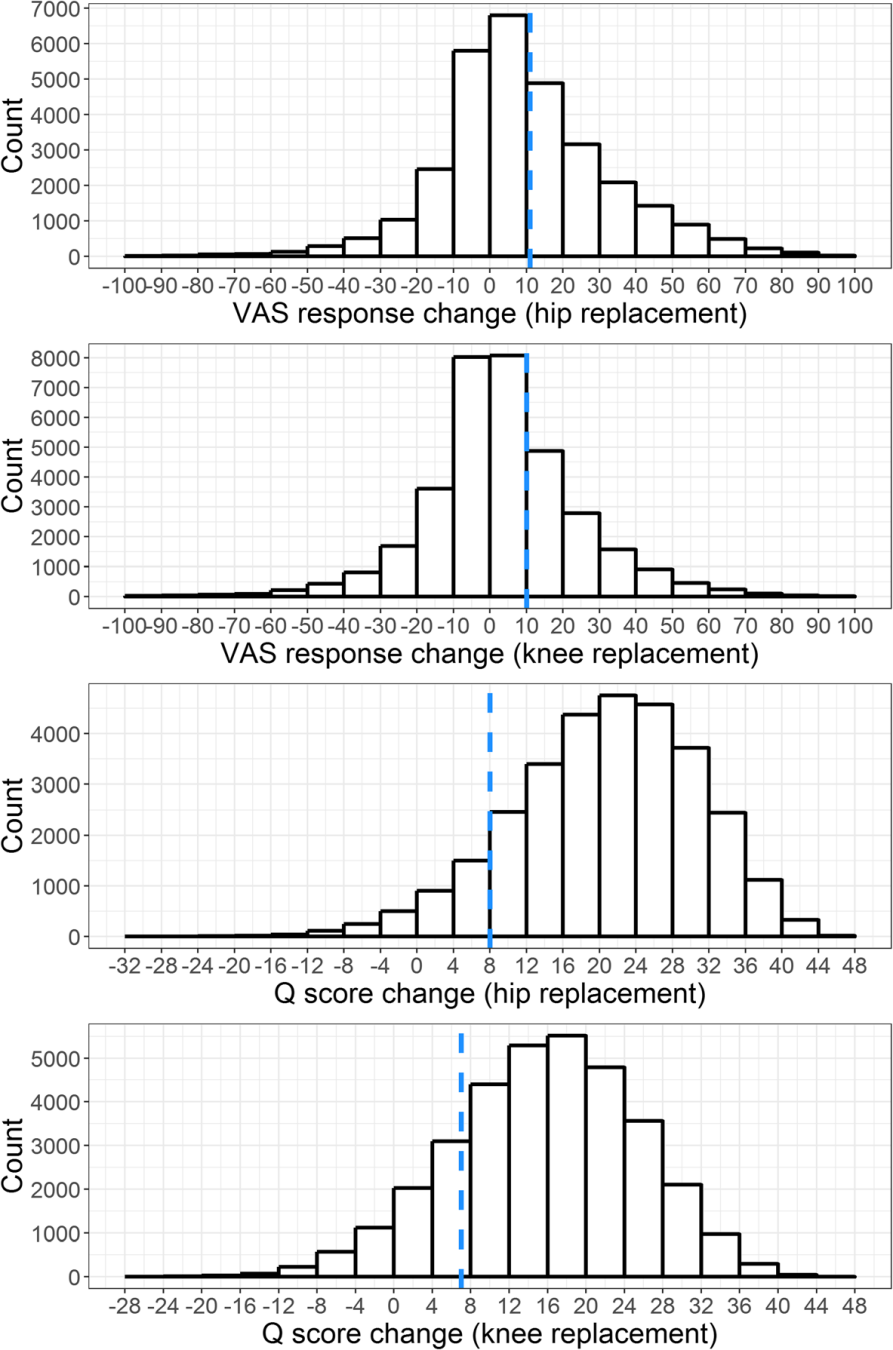
Histograms for postoperative outcome change, VAS and Q score, both procedures, training data. Note: MID depicted as dashed blue line

The histogram (Fig. 1) illustrates postoperative changes (postoperative response minus preoperative response) for both outcomes and procedures. The blue, dashed lines depict MIDs. Outcomes are distributed widely and while only a minority of patients have VAS improvements ranging above MIDs, a clear majority of patients perceive relevant improvements of Q scores.

**Fig. 2:**
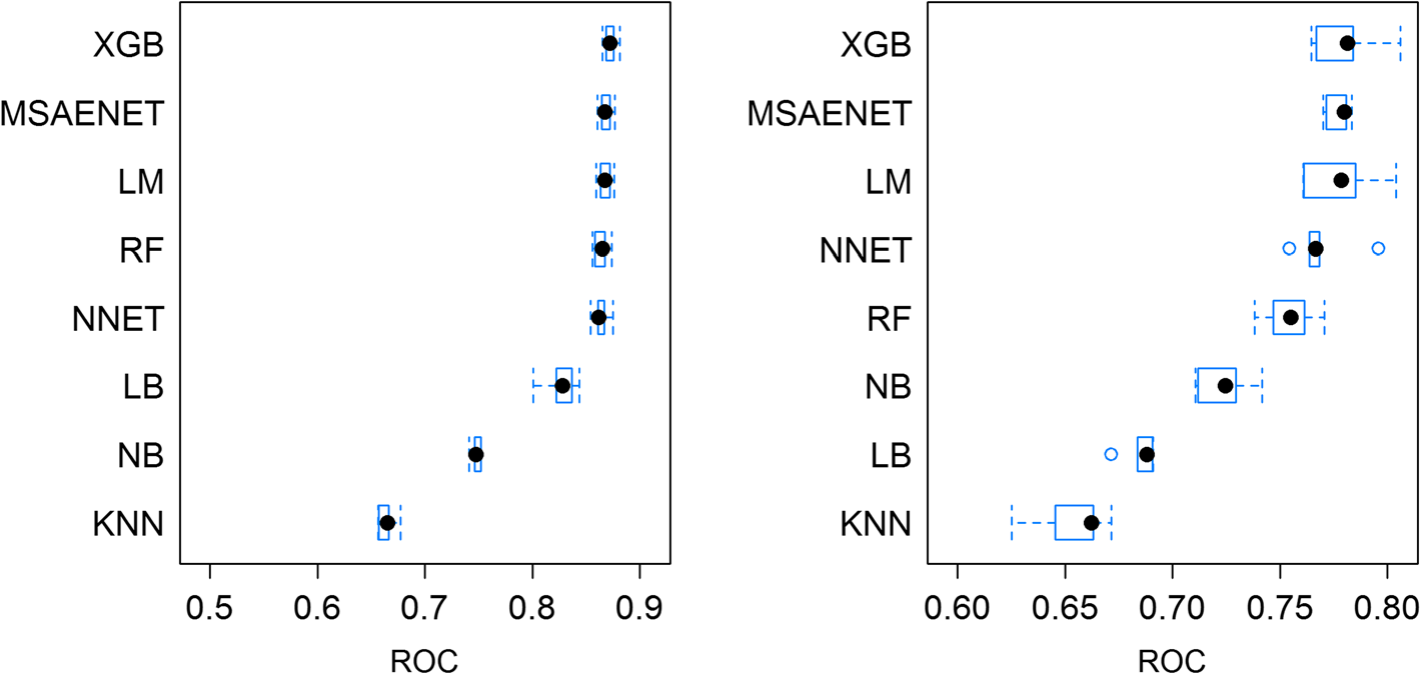
Boxplots, training results (AUROC), postoperative VAS (left) and Q score (right), all models, hip replacement surgery. Note: Outliers depicted as blue dots. XGB: Extreme gradient boosting, MSAENET: Multi-step elastic-net, LM: Linear model, RF: Random forests, NNET: Neural net, LB: Logistic boost, NB: Naïve Bayes, KNN: K-nearest neighbors

Box plots of model performance (Fig. 2) depict AUROC for the VAS and Q score prediction models following hip replacement. For both outcomes, extreme gradient boosting delivered the best AUROC (0.87; 0.78). However, other models followed closely, especially the multistep elastic net and the linear model. Overall, models had higher predictive performance for VAS results than for Q score. Model outcome variation was lower for VAS results. K-Nearest Neighbors had the lowest AUROC for both evaluations.

**Fig. 3:**
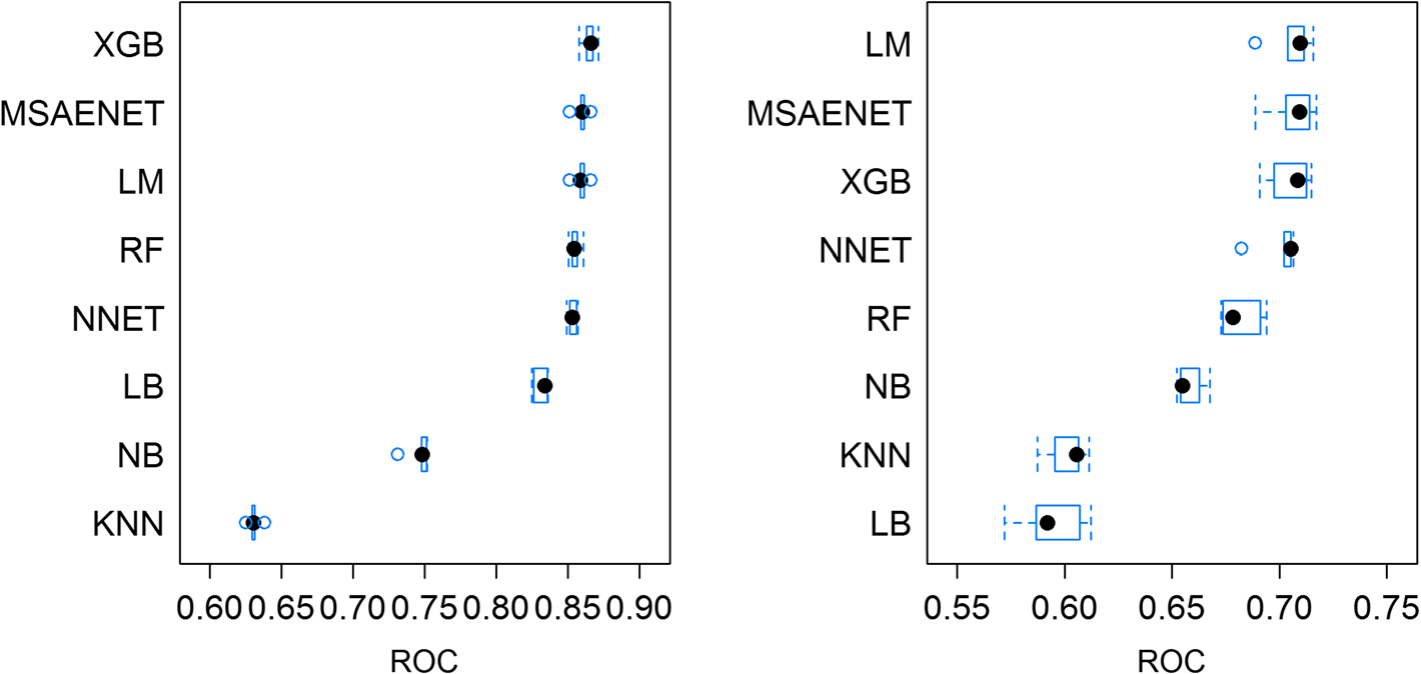
Boxplots, training results (AUROC), postoperative VAS (left) and Q score (right), all models, knee replacement surgery. Note: Outliers depicted as blue dots; XGB: Extreme gradient boosting, MSAENET: Multi-step elastic-net, LM: Linear model, RF: Random forests, NNET: Neural net, LB: Logistic boost, NB: Naïve Bayes, KNN: K-nearest neighbors

The AUROC of VAS models following knee replacement (Fig. 3) were slightly lower compared with the respective hip models. Extreme gradient boosting, multistep elastic net and the linear model delivered the highest median AUROC and were closely trailed by random forest and neural net, which had an AUROC of around 0.83. Linear model, multistep elastic net and extreme gradient boosting had the highest median AUROC (0.71) for post-operative Q score.

Table 2 depicts key performance metrics of the three models with the highest J-statistic for each outcome. The optimal probability thresholds to maximize J-statistic ranged between 0.45 and 0.55. The highest validated J-statistic for each outcome was 0.59 (hip VAS), 0.42 (hip Q score), 0.57 (knee VAS) and 0.31 (knee Q score). Across both procedures and both outcomes, extreme gradient boosting delivered the highest J-statistic, while multistep elastic net, neural net and the linear model followed closely. Among the three models with the highest J-statistic, extreme gradient boosting delivered the highest or equally good F1 scores as well as balanced accuracy as the second best model. Overall, the performance margin was very small and it was easier to predict VAS than Q score improvement, especially for knee replacement surgery. An overview of all performance metrics for all eight models can be found in Additional file 1.

**Table 2:** Key performance metrics of the best three models based on J-statistic, all outcomes

	Hip replacement surgery						Knee replacement surgery					
	VAS			Q score			VAS			Q score		
*Model*	xgbTree	msaenet	neural net	xgbTree	msaenet	glm	xgbTree	msaenet	glm	xgbTree	msaenet	glm
*Training*												
*AUC*	0.87	0.87	0.86	0.78	0.78	0.78	0.87	0.86	0.86	0.71	0.71	0.71
*Best threshold*	0.5	0.45	0.55	0.5	0.5	0.5	0.5	0.45	0.45	0.5	0.5	0.5
*Sensitivity*	0.81	0.79	0.81	0.78	0.76	0.76	0.82	0.79	0.79	0.70	0.69	0.69
*Specificity*	0.76	0.77	0.76	0.64	0.67	0.67	0.73	0.76	0.76	0.59	0.61	0.61
*J-statistic*	0.57	0.57	0.57	0.42	0.43	0.43	0.56	0.56	0.56	0.29	0.30	0.30
*Testing*												
*Sensitivity*	0.82	0.72	0.84	0.79	0.78	0.77	0.83	0.70	0.71	0.70	0.70	0.70
*Specificity*	0.77	0.85	0.73	0.63	0.64	0.65	0.73	0.83	0.83	0.61	0.61	0.62
*Pos Pred Value*	0.75	0.79	0.72	0.96	0.96	0.96	0.62	0.69	0.68	0.91	0.91	0.91
*Neg Pred Value*	0.84	0.78	0.85	0.23	0.22	0.22	0.89	0.85	0.85	0.27	0.27	0.27
*F1*	0.78	0.75	0.78	0.86	0.86	0.85	0.71	0.69	0.69	0.79	0.79	0.79
*Balanced Accuracy*	0.79	0.78	0.79	0.71	0.71	0.71	0.78	0.77	0.77	0.66	0.66	0.66
*J-statistic*	0.59	0.56	0.58	0.42	0.42	0.42	0.57	0.54	0.54	0.31	0.31	0.31

**Fig. 4:**
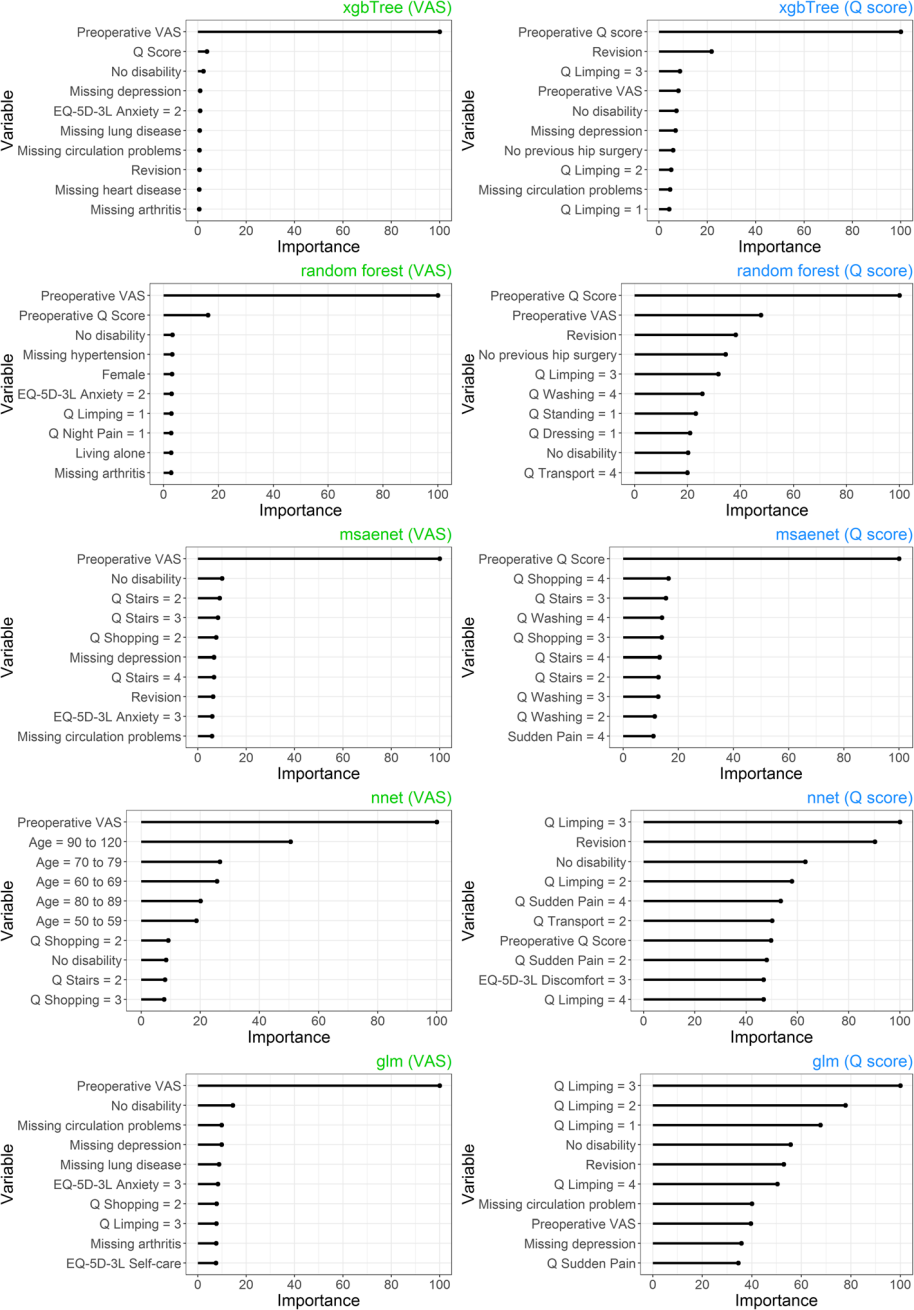
Needle plot of scaled variable importance, several models, hip replacement surgery, top ten variables. Note: Importance does not indicate absolute effect or direction

Figure 4 illustrates variable importance of several models for hip replacement surgery and both outcomes. Preoperative VAS is the most important predictor for postoperative VAS. Preoperative Q score and Q score dimensions, especially the limping question, were the most important predictors for postoperative Q score respectively. Neural net and linear model show greater reliance on dimensional variables.

**Fig. 5:**
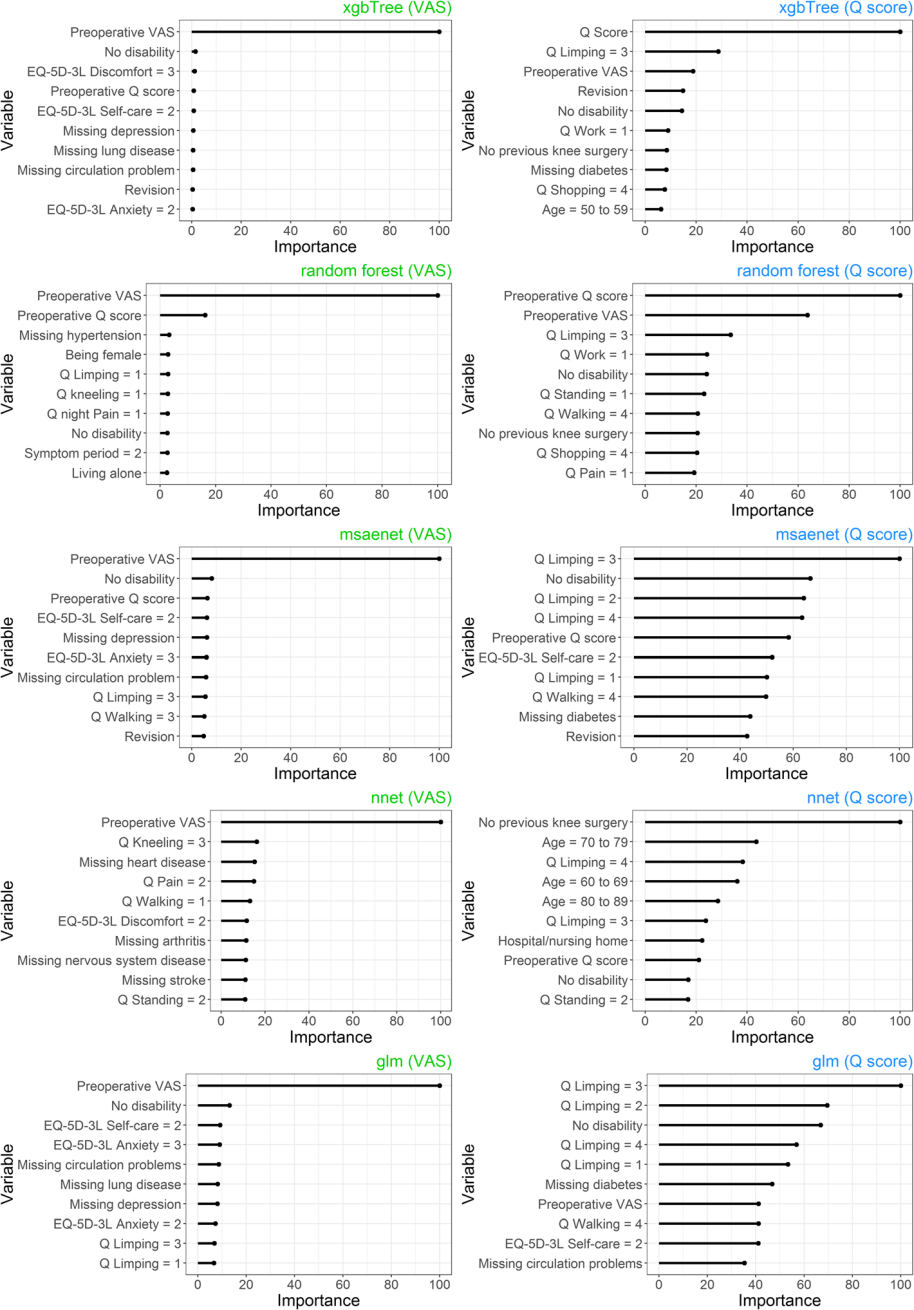
Needle plots of scaled variable importance, VAS and Q score, knee replacement surgery, top ten variables. Note: Importance does not indicate absolute effect or direction

Figure 5 depicts the variable importance of several models for knee replacement surgery and both outcomes. Again, preoperative VAS, preoperative Q score and Q score dimensions, especially the limping question, were the most important variables for each outcome respectively.

## Discussion

This evaluation unveiled three main findings. First, extreme gradient boosting, linear model, multistep elastic net and neural net delivered the highest J-statistic and thus, represent the most robust real world benchmark for one year hip and knee PRO. Second, preoperative VAS, Q score and Q score dimensions were the most important predictors for each respective outcome. Third, it is easier to predict generic VAS than disease-specific Q score and it is easier to predict hip Q score than it is to predict knee Q score.

### Predictive performance and adaptability

The performance margin between the top models was small but extreme gradient boosting delivered the highest overall J-statistic for the four prediction tasks. Extreme gradient boosting is a very versatile algorithm that has been found to perform very well in different machine learning challenges [54]. Its high predictive performance has also been documented for other clinical prediction scenarios like in hip fractures [55], urinary tract infections [56], imaging-based infarcts [57], bioactive molecules [58] and quantitative structure-activity relationships [59]. Due to the ease of implementation and relatively low computing times, compared with other machine learning algorithms, extreme gradient boosting can serve as an alternative to traditional methods or as benchmarking instrument. For our data, the NHS model delivers a sensitivity of 0.77 and a specificity of 0.80. Our extreme gradient boosting model delivers a sensitivity of 0.82 and a specificity of 0.77 (J-statistic 0.57 vs. 0.59). For hip Q scores the extreme gradient boosting model also outperforms the NHS predictions for sensitivity but not specificity (Sensitivity: 0.44 vs. 0.79; specificity: 0.77 vs. 0.63). However, the J-statistic difference is significantly higher (0.21 vs. 0.41). In a next step, we calculated an extreme gradient boosting regression model for the respective data via 10-fold cross validation (3 repetitions). It outperformed the linear model regarding RMSE (16.10 vs. 16.26 for VAS and 7.61 vs. 7.79 for OHS) and MAE (VAS: 11.89 vs. 12.25; OHS: 5.75 vs. 6.15). Overall, despite only incorporating a restrictive set of variables, our model performs slightly better than the predictions provided in the NHS datasets. This confirms robustness of our models.

Extreme gradient boosting provides several hyperparameters (eta, max_depth, colsample_bytree, subsample, nrounds) that can be tuned to improve model performance. Since we only used the standard grid search parameters, performance gains are still possible. Naïve Bayes and KNN delivered only relative low J-statistics. The Naïve Bayesian classifier tended strongly towards sensitivity for all outcomes (0.99, 0.83, 0.99, and 0.87) but had reduced specificity. Decision makers should be aware that utility, cost or loss functions are needed to optimize models for most clinical scenarios and that blindly following AUROC results or J-statistics does not guarantee finding the best classifier for each respective task. Assuming a patient has severe knee or hip pain, suffers from very low HRQoL and, to allow further simplification, only has one opportunity for respective surgery. In this case, prediction models should avoid false negatives and maximize sensitivity, since a patient who greatly benefits from surgery but is predicted not to do so, will suffer significantly from this decision (assuming the surgery decision is based on the prediction), especially when surgery is only possible now but not in the future. However, the easiest way to avoid false negatives is to maximize sensitivity by always predicting improvement for all patients (sensitivity = 100%, false negatives = 0%), irrespective of actual outcome. This is not realistic for most clinical scenarios however, because a high number of false positives is normally associated with risks (e.g. postoperative disability), disutilities and losses. Consequential, sensitivity and specificity should not be viewed alone. Patient and doctor preferences as well as the surgery situation have to be accounted for before model selection.

Speaking more broadly, outcome valuation depends on aims and risk attitude of the patient, in assuring that improvements are being achieved, or deterioration or lack of change are being avoided. The advantage of machine learning is that different algorithms or implementations can deliver higher predictive performance than traditional methods. While machine learning excels at handling huge amounts of predictors and combining them in non-linear, interactive ways [60, 61], linear models may still be a practical option for restrictive data with linear relationships between variables. By using more versatile, non-linear patient data, performance metrics of respective machine learning models will likely improve. It should be noted that for a comparable analysis with longer follow-up periods and less restrictive data with more variables, computing time will increase superlinearly. Hardware needs should therefore be accounted for. Since we only used the standard grid search approach, performance gains are still possible, by fine-tuning associated hyperparameters. Additional training years will also lead to better predictive performance.

### Variable importance

Many machine-learning algorithms can reach very high predictive performance but don’t solve the problem of causal inference. However, both, traditional methods and machine learning, point us towards meaningful medical conclusions [62]. For example, when overweight is of high importance, doctors may counsel patients to lose weight. While it would be desirable to understand the underlying principles and causative variables of perfect prediction models, it is no requirement to use respective models for SDM. The prediction itself provides inherent value by supplementing available evidence. While inference and machine learning are often viewed as separated entities, variable importance of machine learning classifiers is used for the evaluation of a wide variety of different research objectives. They include healthcare spending [63], identification of biomarkers for knee osteoarthritis [64], microarray studies [65], credit default risk of enterprises [66], energy performance of buildings [67] or even landslide susceptibility modeling [68]. By providing the variable importance of five different models, we illustrated the predictive importance of preoperative VAS and Q score as well as respective dimensions. Vogl et al. 2014 [69] and other studies [70, 71] confirm the importance of preoperative HRQoL for postoperative HRQoL. The likely reason is that patients with low preoperative HRQoL can benefit significantly from respective surgery, while patients with high preoperative HRQoL cannot or can only improve slightly. The university of York developed an informed clinical decision tool to predict improvement for hip and knee replacement surgery that also strongly relies on preoperative EQ-5D-3L index as well as age, gender and symptom duration [72]. The Pearson correlation for preoperative and postoperative hip VAS, hip Q score, knee VAS, knee Q score was 0.33, 0.30, 0.40 and 0.39 respectively. This indicates a moderate correlation. For testing purposes, we calculated the AUROC for extreme gradient boosting and all outcomes by only using preoperative VAS or Q score. The highest AUROCs for hip and knee VAS were 0.85 and 0.82 (preoperative VAS only), compared with 0.87 and 0.87 for all variables. As indicated by Figs. 4 and 5, predictive performance for Q score is more reliant on multivariate data. To validate this finding we calculated univariate AUROCs by using preoperative Q score only. This yielded maximum AUROCs of 0.69 (univariate preoperative hip Q score) and 0.62 (univariate preoperative knee Q score), while the original multivariate AUROCs were 0.78 and 0.71 respectively. This wider difference confirms that Q score models are less reliant on the preoperative Q score and require additional variables to reach optimal results. It should be noted that variable importance can be calculated in different ways for different models. Some methods, under specific circumstances and especially for random forests, can introduce bias and artificial variable selection, while random permutations can cause additional issues [73, 74]. However, the ranking of our top variables was constant among different runs, we included several different models with different methods and univariate analysis confirmed their importance.

### Differences between hip and knee replacement

Compared with the average knee replacement surgery patient, the average hip replacement surgery patient has lower preoperative VAS (64.85 vs. 68.18) and Q score (18.47 vs. 19.34) but also has better improvement following surgery (+ 12.06 vs. + 6.09 for VAS and + 21.19 vs. 16.26 for Q score). 6.09 is below our respective MID, meaning that the average patient does not reach relevant generic improvement. However, average Q score change is significantly above the respective MID, indicating that relevant disease-specific improvement is present following surgery. Thus, the choice of HRQoL instrument has significant influence on outcome achievement. Greater improvement with hip replacement falls in line with other research [75, 76] and is likely based on the greater complexity of knee replacement surgery. We also showed that predicting VAS results (AUROC of around 0.87 for hip and 0.87 for knee) is easier than predicting Q scores (AUROC of around 0.78 for hip and 0.70 for knee). One explanation for this difference is the nature of both instruments. VAS results represent a generic summary of health perception and consequentially should be less sensitive to disease-specific influences, as shown by our evaluation. Despite ranging from 0 to 100, VAS results on average, only improve 6 and 12 points, while Q scores, ranging only from 0 to 48, improve by 16 and 21 points respectively. Nevertheless, VAS outcomes represent a more holistic approach that may account for aspects of disease, which are not directly addressed via disease-specific instruments.

### Clinical relevance

One important way to support shared decision-making is to provide patients and doctors with highly accurate prediction models for relevant outcomes. From a patient perspective, relevant outcomes in osteoarthritis include HRQoL as well as contextual barriers, treatment disadvantages and consequences for personal life [77]. Our evaluation focused on HRQoL, since it resembles an overall aggregate of patient health perception. When clinicians want to predict postoperative HRQoL, they can rely on either personal expertise, average patient results or individual prediction models. These prediction models should incorporate significant numbers of population-based surgery observations from a real-world context in order to be representative. Our models incorporate data of over 60,000 recent hip and knee replacement surgeries from a real world, routine care, population-based registry and we apply different algorithms/implementations to reach high predictive performance. By delivering real-world benchmarks, results from our models supplement clinical expertise and thus, may contribute to shared-decision making. Clinicians should be aware that predictive performance of our models can be improved further by using more detailed clinical data (e.g. ASA class, blood values, BMI etc.) that were not available for the conduct of this study but that are typically gathered before elective surgery, also on a routine basis. We further showed that preoperative PROMs are the most important predictors for postoperative PROMs. The underlying PROMs can be gathered easily in clinical settings on a routine basis though limitations do exist [78]. The two small self-explanatory surveys are filled out in a few minutes or less and do not require any previous knowledge by the patient.

Another aspect of clinical relevance of this study is that PROMs-based quality of care improvement requires defined standards on postoperative PROMs change [79]. By providing individual outcome estimations, we deliver a more (VAS) or less (Q score) reliable standard to incorporate PROMs into clinical quality of care control.

### Sensitivity analysis

Different methods exist to calculate MIDs. To evaluate the influence of MID on model performance we conducted several univariate sensitivity analyses, in a first example, for hip VAS patients. Since MID selection influences the proportion of patients who can achieve MID-based improvement, we also tested the influence of removing respective patients from the dataset. A patient with preoperative VAS score of 90 is not able to achieve postoperative gains greater than 10. Thus, selecting higher MIDs results in less patients being able to achieve improvement, supposedly making it easier for models to predict the correct outcome by only incorporating preoperative VAS score. Our first sensitivity analysis (Additional file 2) concerned patients with hip replacement and tested a MID of 23 for EQ-5D-3L VAS that was stated in a Danish study by Paulsen et al. 2014 [52]. This improved the AUROCs of the best five models to 0.91/0.92 (compared with 0.86/0.87 before; MID = 11). This gain is not surprising, since significantly less patients can achieve this MID. Removing all patients not able to achieve MID, reduced respective AUROCs to 0.83/0.84 for the best five models (Additional file 3) and reduced the number of observations to 19,716. Taking the example of our main evaluation and filtering all patients who could not achieve a VAS MID of 11 resulted in 25,606 remaining observations and AUROCs of the best models ranging around 0.81/0.82 (Additional file 4). MID selection, filtering of patients and number of observations all have significant influence on model performance.

### Limitations

Strengths of this study include the wide variety of algorithms that were applied for evaluation as well as the testing of specific probability thresholds to find the best classifier. By reporting the J-statistic, we go beyond AUROC calculation and show maximal performance when sensitivity and specificity are valued the same. Moreover, the incorporation of generic and disease-specific outcomes for both, hip and knee replacement surgery, gave insights for both instruments and both procedures.

One limitation of this study is the lack of controls. It was not possible to model patient trajectories without surgery. It is unknown, if a patient has no improvement because of surgery or if surgery prevented an otherwise significant deterioration of health outcome. The lack of long-term data made it impossible to make long-term predictions. Some patients will only have temporary improvement and long-term data are needed to evaluate this issue. Moreover, we only evaluated a binary outcome (improvement/no improvement) but patients may want to know the degree of improvement or deterioration. This could be investigated in future research but results and associated uncertainty are more difficult to apply in shared decision-making. We had no utility, loss or cost function to optimize model metrics because costs were not available and utilities change by patient. Due to privacy concerns, public NHS PROMs data are restrictive and do not reflect clinical precision and versatility. For example, age bands in NHS data cover 10-year time spans and other variables like rehabilitation, BMI or allergies, despite having been found to influence knee and hip replacement outcomes [80–83], are completely missing. Incorporating respective data will likely improve predictive performance of models. Furthermore, between pre- and postoperative patient reports, response shift has been observed in the UK PROMs data which potentially reduces patient’s gain but could not further be analyzed here [84]. Conflicting evidence regarding the validity of self-reported patient data exists [85, 86]. However, a rigorous recent study concluded that patient reporting provides similar and less costly information compared with medical records [87]. Moreover, comorbidities in hospital medical records are often based on self-report as well, since clinical validation is mostly not feasible. When we ensembled all models linearly for both procedures and both outcomes (not shown here), the resulting AUROC was either worse or only minimally better (third decimal place) than for single models alone. Ensembling of different models was not the focus of this study and thus, we refrained from adding additional uncertainty.

## Conclusion

We provide robust real world benchmarking results for the prediction of PROMs-based postoperative hip and knee replacement surgery outcomes. Extreme gradient boosting delivered the highest overall J-statistic among all models. Linear model, multistep elastic net and neural net followed closely. One strength of machine learning models is their adaptability to different clinical scenarios where certain levels of sensitivity or specificity are needed. Preoperative VAS, Q score and specific instrument dimensions like lumping, were the most important predictors for hip and knee replacement surgery PROMs.

## Additional files

Additional file 1: Performance metrics including J-statistic, training and test set, both procedures, all models. (DOCX 19 kb)

Additional file 2: Univariate increase of VAS MID to 23, hip replacement results (AUROC, Sensitivity, Specificity), no filtering. (TIFF 8437 kb)

Additional file 3: Univariate increase of VAS MID to 23, hip replacement results (AUROC, Sensitivity, Specificity), filtering impossible improvement (remaining *n* = 19,716). (TIFF 8437 kb)

Additional file 4: Univariate increase of VAS MID to 11, hip replacement results (AUROC, Sensitivity, Specificity), filtering impossible improvement (remaining *n* = 25,606). (TIFF 8437 kb)

## References

[1] Elwyn G, Frosch D, Thomson R (2012). Shared decision making: a model for clinical practice. J Gen Intern Med.

[2] Oshima Lee E, Emanuel EJ (2013). Shared decision making to improve care and reduce costs. N Engl J Med.

[3] Stacey D, Légaré F, Lewis K, et al. Decision aids for people facing health treatment or screening decisions. Cochrane Database of Systematic Reviews. 2017. 10.1002/14651858.CD001431.pub5 PMC6478132 28402085

[4] Wagle NW. Care Redesign 2016: Implementing Patient-Reported Outcome Measures. NEJM Catalyst:2016.

[5] Devlin NJ, Appleby J (2010). Getting the most out of PROMS.

[6] Nilsson E, Orwelius L, Kristenson M (2016). Patient-reported outcomes in the Swedish National Quality Registers. J Intern Med.

[7] Baumhauer JF (2017). Patient-reported outcomes — are they living up to their potential?. N Engl J Med.

[8] Eneqvist T, Nemes S, Bulow E (2018). Can patient-reported outcomes predict re-operations after total hip replacement?. Int Orthop.

[9] ShahabiKargar Z, Khanna S, Good N (2014). Predicting procedure duration to improve scheduling of elective surgery.

[10] Kargar ZS, Khanna S, Sattar A (2013). Using prediction to improve elective surgery scheduling. Australas Med J.

[11] Wong DJN, Oliver CM, Moonesinghe SR (2017). Predicting postoperative morbidity in adult elective surgical patients using the surgical outcome risk tool (SORT). Br J Anaesth.

[12] Moonesinghe SR, Mythen MG, Das P (2013). Risk stratification tools for predicting morbidity and mortality in adult patients undergoing major SurgeryQualitative systematic review. Anesthesiology.

[13] National Joint Registry. Joint replacement statistics. 2017.

[14] Miguel-Hurtado O, Guest R, Stevenage SV (2016). Comparing machine learning classifiers and linear/logistic regression to explore the relationship between Hand dimensions and demographic characteristics. PLoS One.

[15] Seligman B, Tuljapurkar S, Rehkopf D (2018). Machine learning approaches to the social determinants of health in the health and retirement study. SSM - Population Health.

[16] Singal AG, Mukherjee A, Elmunzer BJ (2013). Machine learning algorithms outperform conventional regression models in predicting development of hepatocellular carcinoma. Am J Gastroenterol.

[17] Rigg J, Lodhi H, Nasuti P (2015). PRM130 - using machine learning to detect patients with undiagnosed rare diseases: an application of support vector machines to a rare oncology disease. Value Health.

[18] Chen JH, Asch SM (2017). Machine learning and prediction in medicine — beyond the peak of inflated expectations. N Engl J Med.

[19] Wolpert DH (1996). The lack of a priori distinctions between learning algorithms. Neural Comput.

[20] Wolpert DH (1995). Macready WG.

[21] L’Heureux A, Grolinger K, Elyamany HF (2017). Machine learning with big data: challenges and approaches. IEEE Access.

[22] Luo G (2016). PredicT-ML: a tool for automating machine learning model building with big clinical data. Health Inf Sci Syst.

[23] Deo RC (2015). Machine learning in medicine. Circulation.

[24] National Health Service. Patient Reported Outcome Measures (PROMs). 2018.

[25] EuroQol--a new facility for the measurement of health-related quality of life. Health policy. 1990;16(3):199-208. 10.1016/0168-8510(90)90421-9 10109801

[26] Dawson J, Fitzpatrick R, Carr A (1996). Questionnaire on the perceptions of patients about total hip replacement. J Bone Joint Surg Br.

[27] Dawson J, Fitzpatrick R, Murray D (1998). Questionnaire on the perceptions of patients about total knee replacement. J Bone Joint Surg Br..

[28] Chawla NV, Bowyer KW, Hall LO (2002). SMOTE: synthetic minority over-sampling technique. J Artif Intell Res.

[29] Batista GEAPA, Prati RC, Monard MC (2004). A study of the behavior of several methods for balancing machine learning training data. SIGKDD Explor Newsl.

[30] Thornton C, Hutter F, Hoos HH (2013). Auto-WEKA: combined selection and hyperparameter optimization of classification algorithms. Proceedings of the 19th ACM SIGKDD international conference on knowledge discovery and data mining.

[31] Kuhn M (2008). Caret package. J Stat Softw.

[32] Ericson G, Rohm WA, et al. Machine learning algorithm cheat sheet for Azure Machine Learning Studio. Microsoft. 2017. https://docs.microsoft.com/en-us/azure/machine-learning/studio/algorithm-cheat-sheet. Accessed 30 Dec 2018.

[33] Li H. Which machine learning algorithm should I use? The SAS Data Science Blog. 2017. https://blogs.sas.com/content/subconsciousmusings/2017/04/12/machine-learning-algorithm-use. Accessed 30 Dec 2018.

[34] scikit-learn developers. Choosing the right estimator. scikit-learn. 2017. https://scikit-learn.org/stable/tutorial/machine_learning_map/index.html. Accessed 30 Dec 2018.

[35] Sauer S, Buettner R, Heidenreich T (2018). Mindful machine learning. Eur J Psychol Assess.

[36] Chen T, Guestrin C (2016). XGBoost: a scalable tree boosting system. Proceedings of the 22nd ACM SIGKDD international conference on knowledge discovery and data mining.

[37] Xiao N, Xu Q-S (2015). Multi-step adaptive elastic-net: reducing false positives in high-dimensional variable selection. J Stat Comput Simul.

[38] Liaw A, Wiener M (2001). Classification and regression by RandomForest.

[39] Kleene SC. Representation of events in nerve nets and finite automata. Rand project air force Santa Monica CA. 1951. https://apps.dtic.mil/dtic/tr/fulltext/u2/a596138.pdf. Accessed 30 Dec 2018.

[40] Haykin S (1998). Neural networks: a Comprehensive Foundation.

[41] Hand DJ, Yu K. Idiot's Bayes: not so stupid after all? International Statistical Review. 2001;69(3):385–98.

[42] Cover T, Hart P (1967). Nearest neighbor pattern classification. IEEE Trans Inf Theory.

[43] Friedman J, Hastie T, Tibshirani R (2000). Additive logistic regression: a statistical view of boosting (with discussion and a rejoinder by the authors). Ann Stat.

[44] Steinwart I, Christmann A (2008). Support vector machines.

[45] Bradley AP (1997). The use of the area under the ROC curve in the evaluation of machine learning algorithms. Pattern Recogn.

[46] Youden WJ (1950). Index for rating diagnostic tests. Cancer.

[47] Kuhn M. Variable importance using the caret package.

[48] NHS Digital. Patient reported outcome measures (PROMs) in England - a guide to PROMs methodology. NHS Digital. 2017. https://digital.nhs.uk/binaries/content/assets/legacy/pdf/g/t/proms_guide_v12.pdf. Accessed 30 Dec 2018.

[49] King MT (2011). A point of minimal important difference (MID): a critique of terminology and methods. Expert Rev Pharmacoecon Outcomes Res.

[50] Revicki D, Hays RD, Cella D (2008). Recommended methods for determining responsiveness and minimally important differences for patient-reported outcomes. J Clin Epidemiol.

[51] Norman GR, Sloan JA, Wyrwich KW (2003). Interpretation of changes in health-related quality of life: the remarkable universality of half a standard deviation. Med Care.

[52] Paulsen A, Roos EM, Pedersen AB (2014). Minimal clinically important improvement (MCII) and patient-acceptable symptom state (PASS) in total hip arthroplasty (THA) patients 1 year postoperatively. Acta Orthop.

[53] Beard DJ, Harris K, Dawson J (2015). Meaningful changes for the Oxford hip and knee scores after joint replacement surgery. J Clin Epidemiol.

[54] Nielsen D. Tree Boosting With XGBoost-Why Does XGBoost Win" Every" Machine Learning Competition? Master's thesis, NTNU. 2016. https://brage.bibsys.no/xmlui/bitstream/handle/11250/2433761/16128_FULLTEXT.pdf. Accessed 30 Dec 2018.

[55] Kruse C, Eiken P, Vestergaard P (2017). Machine learning principles can improve hip fracture prediction. Calcif Tissue Int.

[56] Taylor RA, Moore CL, Cheung KH (2018). Predicting urinary tract infections in the emergency department with machine learning. PLoS One.

[57] Livne M, Boldsen JK, Mikkelsen IK (2018). Boosted tree model reforms multimodal magnetic resonance imaging infarct prediction in acute stroke. Stroke.

[58] Babajide Mustapha I, Saeed F. Bioactive molecule prediction using extreme gradient boosting. Molecules. 2016;21. 10.3390/molecules21080983 PMC6273295 27483216

[59] Sheridan RP, Wang WM, Liaw A (2016). Extreme gradient boosting as a method for quantitative structure-activity relationships. J Chem Inf Model.

[60] Mullainathan S, Spiess J (2017). Machine learning: an applied econometric approach. J Econ Perspect.

[61] Obermeyer Z, Emanuel EJ (2016). Predicting the future — big data, machine learning, and clinical medicine. N Engl J Med.

[62] Bzdok D, Altman N, Krzywinski M. Points of significance: statistics versus machine learning. Nat Methods. 2018:1–7. 10.1038/nmeth.4642 PMC6082636 30100822

[63] Rose S. Robust machine learning variable importance analyses of medical conditions for health care spending. Health Serv Res. 2018. 10.1111/1475-6773.12848 PMC6153184 29527659

[64] Lazzarini N, Runhaar J, Bay-Jensen AC (2017). A machine learning approach for the identification of new biomarkers for knee osteoarthritis development in overweight and obese women. Osteoarthr Cartil.

[65] Archer KJ, Kimes RV (2008). Empirical characterization of random forest variable importance measures. Comput Stat Data Anal.

[66] Yao J, Levy-Chapira M, Margaryan M. Checking account activity and credit default risk of enterprises: An application of statistical learning methods. arXiv. 2017. preprint arXiv:1707.00757.

[67] Tsanas A, Xifara A (2012). Accurate quantitative estimation of energy performance of residential buildings using statistical machine learning tools. Energ Buildings.

[68] Goetz J, Brenning A, Petschko H (2015). Evaluating machine learning and statistical prediction techniques for landslide susceptibility modeling. Comput Geosci.

[69] Vogl M, Wilkesmann R, Lausmann C (2014). The impact of preoperative patient characteristics on health states after total hip replacement and related satisfaction thresholds: a cohort study. Health Qual Life Outcomes.

[70] Schilling CG, Dowsey MM, Petrie DJ (2017). Predicting the Long-Term Gains in Health-Related Quality of Life After Total Knee Arthroplasty. J Arthroplasty.

[71] Sprague S, Bhandari M, Heetveld MJ (2018). Factors associated with health-related quality of life, hip function, and health utility after operative management of femoral neck fractures. Bone Joint J.

[72] Gutacker N, Street A (2017). Use of large-scale HRQoL datasets to generate individualised predictions and inform patients about the likely benefit of surgery. Qual Life Res.

[73] Strobl C, Boulesteix A-L, Zeileis A, et al. Bias in random forest variable importance measures: illustrations, sources and a solution. BMC Bioinformatics. 2007;8:25–5. 10.1186/1471-2105-8-25 PMC1796903 17254353

[74] Liaw A, Wiener M (2002). Classification and regression by randomForest. R news.

[75] Mandzuk LL, McMillan DE, Bohm ER (2015). A longitudinal study of quality of life and functional status in total hip and total knee replacement patients. International journal of orthopaedic and trauma nursing.

[76] Liebs TR, Herzberg W, Ruther W (2016). Quality-adjusted life years gained by hip and knee replacement surgery and its aftercare. Arch Phys Med Rehabil.

[77] Selten EM, Geenen R, van der Laan WH (2017). Hierarchical structure and importance of patients' reasons for treatment choices in knee and hip osteoarthritis: a concept mapping study. Rheumatology (Oxford).

[78] Feng Y, Parkin D, Devlin NJ (2014). Assessing the performance of the EQ-VAS in the NHS PROMs programme. Qual Life Res.

[79] Prodinger B, Taylor P (2018). Improving quality of care through patient-reported outcome measures (PROMs): expert interviews using the NHS PROMs Programme and the Swedish quality registers for knee and hip arthroplasty as examples. BMC Health Serv Res.

[80] Singh JA, Lewallen D (2009). Age, gender, obesity, and depression are associated with patient-related pain and function outcome after revision total hip arthroplasty. Clin Rheumatol.

[81] Otero JE, Graves CM, Gao Y (2016). Patient-reported allergies predict worse outcomes after hip and knee arthroplasty: results from a prospective cohort study. J Arthroplast.

[82] Xu S, Chen JY, Lo NN (2018). The influence of obesity on functional outcome and quality of life after total knee arthroplasty. Bone Joint J.

[83] Snell DL, Hipango J, Sinnott KA, et al. Rehabilitation after total joint replacement: a scoping study. Disabil Rehabil. 2018;40:1718–31. 10.1080/09638288.2017.1300947 28330380

[84] Pickard AS, Hung YT, Lin FJ (2017). Patient experience-based value sets: are they stable?. Med Care.

[85] Olomu AB, Corser WD, Stommel M, et al. Do self-report and medical record comorbidity data predict longitudinal functional capacity and quality of life health outcomes similarly? BMC Health Serv Res. 2012;12:398–8. 10.1186/1472-6963-12-398 PMC3538524 23151237

[86] van den Akker M, van Steenkiste B, Krutwagen E (2015). Disease or no disease? Disagreement on diagnoses between self-reports and medical records of adult patients. Eur J Gen Pract.

[87] Ye F, Moon DH, Carpenter WR (2017). Comparison of patient report and medical records of comorbidities: results from a population-based cohort of patients with prostate cancer. JAMA Oncology.

