# Supplementary material for: Predicting patient-reported outcomes following hip and knee replacement surgery using supervised machine learning
Source: BMC Med Inform Decis Mak. 2019 Jan 8;19:3. doi: 10.1186/s12911-018-0731-6 (PMC6325823; doi:10.1186/s12911-018-0731-6)
Supplement: Supplementary file 1 — Performance metrics including J-statistic, training and test set, both procedures, all models. (DOCX 19 kb) [file 12911_2018_731_MOESM1_ESM.docx]

|  | ***xgbTree*** | ***msaenet*** | ***glm*** | ***neural net*** | ***random forest*** | ***logit boost*** | ***naive bayes*** | ***knn*** |
| --- | --- | --- | --- | --- | --- | --- | --- | --- |
| **Training (hip VAS)** |  |  |  |  |  |  |  |  |
| Best threshold | 0.5 | 0.45 | 0.45 | 0.55 | 0.45 | 0.5 | 0.9 | 0.55 |
| Sensitivity | 0.81 | 0.79 | 0.79 | 0.81 | 0.81 | 0.81 | 0.91 | 0.62 |
| Specificity | 0.76 | 0.77 | 0.78 | 0.76 | 0.75 | 0.75 | 0.37 | 0.62 |
| J-statistic | 0.57 | 0.57 | 0.57 | 0.57 | 0.56 | 0.53 | 0.29 | 0.24 |
| **Testing (hip VAS)** |  |  |  |  |  |  |  |  |
| Sensitivity | 0.82 | 0.72 | 0.72 | 0.84 | 0.73 | 0.75 | 0.99 | 0.66 |
| Specificity | 0.77 | 0.85 | 0.85 | 0.73 | 0.83 | 0.80 | 0.13 | 0.59 |
| Pos Pred Value | 0.75 | 0.79 | 0.79 | 0.72 | 0.79 | 0.76 | 0.49 | 0.57 |
| Neg Pred Value | 0.84 | 0.78 | 0.78 | 0.85 | 0.79 | 0.79 | 0.97 | 0.67 |
| F1 | 0.78 | 0.75 | 0.75 | 0.78 | 0.76 | 0.76 | 0.65 | 0.61 |
| Balanced Accuracy | 0.79 | 0.78 | 0.78 | 0.79 | 0.78 | 0.78 | 0.56 | 0.62 |
| J-statistic | 0.59 | 0.56 | 0.56 | 0.58 | 0.56 | 0.55 | 0.12 | 0.24 |
| **Training (hip Q score)** |  |  |  |  |  |  |  |  |
| Best threshold | 0.5 | 0.5 | 0.5 | 0.45 | 0.65 | 0.5 | 0.8 | 0.6 |
| Sensitivity | 0.78 | 0.76 | 0.76 | 0.80 | 0.74 | 0.80 | 0.79 | 0.61 |
| Specificity | 0.64 | 0.67 | 0.67 | 0.62 | 0.64 | 0.55 | 0.54 | 0.64 |
| J-statistic | 0.42 | 0.43 | 0.43 | 0.42 | 0.38 | 0.34 | 0.33 | 0.26 |
| **Testing (hip Q score)** |  |  |  |  |  |  |  |  |
| Sensitivity | 0.79 | 0.78 | 0.77 | 0.78 | 0.95 | 0.77 | 0.83 | 0.71 |
| Specificity | 0.63 | 0.64 | 0.65 | 0.63 | 0.30 | 0.56 | 0.49 | 0.54 |
| Pos Pred Value | 0.96 | 0.96 | 0.96 | 0.96 | 0.93 | 0.95 | 0.94 | 0.94 |
| Neg Pred Value | 0.23 | 0.22 | 0.22 | 0.22 | 0.37 | 0.20 | 0.22 | 0.15 |
| F1 | 0.86 | 0.86 | 0.85 | 0.86 | 0.94 | 0.85 | 0.88 | 0.81 |
| Balanced Accuracy | 0.71 | 0.71 | 0.71 | 0.71 | 0.62 | 0.67 | 0.66 | 0.62 |
| J-statistic | 0.42 | 0.42 | 0.42 | 0.41 | 0.25 | 0.33 | 0.32 | 0.25 |
| **Training (knee VAS)** |  |  |  |  |  |  |  |  |
| Best threshold | 0.5 | 0.45 | 0.45 | 0.45 | 0.4 | 0.5 | 0.95 | 0.55 |
| Sensitivity | 0.82 | 0.79 | 0.79 | 0.82 | 0.80 | 0.75 | 0.76 | 0.59 |
| Specificity | 0.73 | 0.76 | 0.76 | 0.72 | 0.75 | 0.78 | 0.59 | 0.61 |
| J-statistic | 0.56 | 0.56 | 0.56 | 0.54 | 0.54 | 0.53 | 0.36 | 0.19 |
| **Testing (knee VAS)** |  |  |  |  |  |  |  |  |
| Sensitivity | 0.83 | 0.70 | 0.71 | 0.71 | 0.55 | 0.76 | 0.99 | 0.63 |
| Specificity | 0.73 | 0.83 | 0.83 | 0.82 | 0.90 | 0.78 | 0.18 | 0.56 |
| Pos Pred Value | 0.62 | 0.69 | 0.68 | 0.67 | 0.74 | 0.64 | 0.38 | 0.42 |
| Neg Pred Value | 0.89 | 0.85 | 0.85 | 0.85 | 0.80 | 0.86 | 0.98 | 0.75 |
| F1 | 0.71 | 0.69 | 0.69 | 0.69 | 0.63 | 0.70 | 0.55 | 0.51 |
| Balanced Accuracy | 0.78 | 0.77 | 0.77 | 0.77 | 0.73 | 0.77 | 0.59 | 0.60 |
| J-statistic | 0.57 | 0.54 | 0.54 | 0.53 | 0.45 | 0.54 | 0.18 | 0.19 |
| **Training (knee Q score)** |  |  |  |  |  |  |  |  |
| Best threshold | 0.5 | 0.5 | 0.5 | 0.55 | 0.65 | 0.5 | 0.9 | 0.45 |
| Sensitivity | 0.70 | 0.69 | 0.69 | 0.63 | 0.63 | 0.80 | 0.80 | 0.62 |
| Specificity | 0.59 | 0.61 | 0.61 | 0.66 | 0.63 | 0.38 | 0.42 | 0.52 |
| J-statistic | 0.29 | 0.30 | 0.30 | 0.29 | 0.26 | 0.17 | 0.22 | 0.15 |
| **Testing (knee Q score)** |  |  |  |  |  |  |  |  |
| Sensitivity | 0.70 | 0.70 | 0.70 | 0.73 | 0.94 | 0.78 | 0.87 | 0.55 |
| Specificity | 0.61 | 0.61 | 0.62 | 0.58 | 0.23 | 0.40 | 0.34 | 0.59 |
| Pos Pred Value | 0.91 | 0.91 | 0.91 | 0.90 | 0.87 | 0.88 | 0.88 | 0.88 |
| Neg Pred Value | 0.27 | 0.27 | 0.27 | 0.28 | 0.40 | 0.25 | 0.32 | 0.19 |
| F1 | 0.79 | 0.79 | 0.79 | 0.81 | 0.90 | 0.83 | 0.87 | 0.68 |
| Balanced Accuracy | 0.66 | 0.66 | 0.66 | 0.65 | 0.59 | 0.59 | 0.60 | 0.57 |
| J-statistic | 0.31 | 0.31 | 0.31 | 0.30 | 0.17 | 0.18 | 0.21 | 0.14 |
