# Supplementary figures and images for: Predicting patient-reported outcomes following hip and knee replacement surgery using supervised machine learning
Source: BMC Med Inform Decis Mak. 2019 Jan 8;19:3. doi: 10.1186/s12911-018-0731-6 (PMC6325823; doi:10.1186/s12911-018-0731-6)

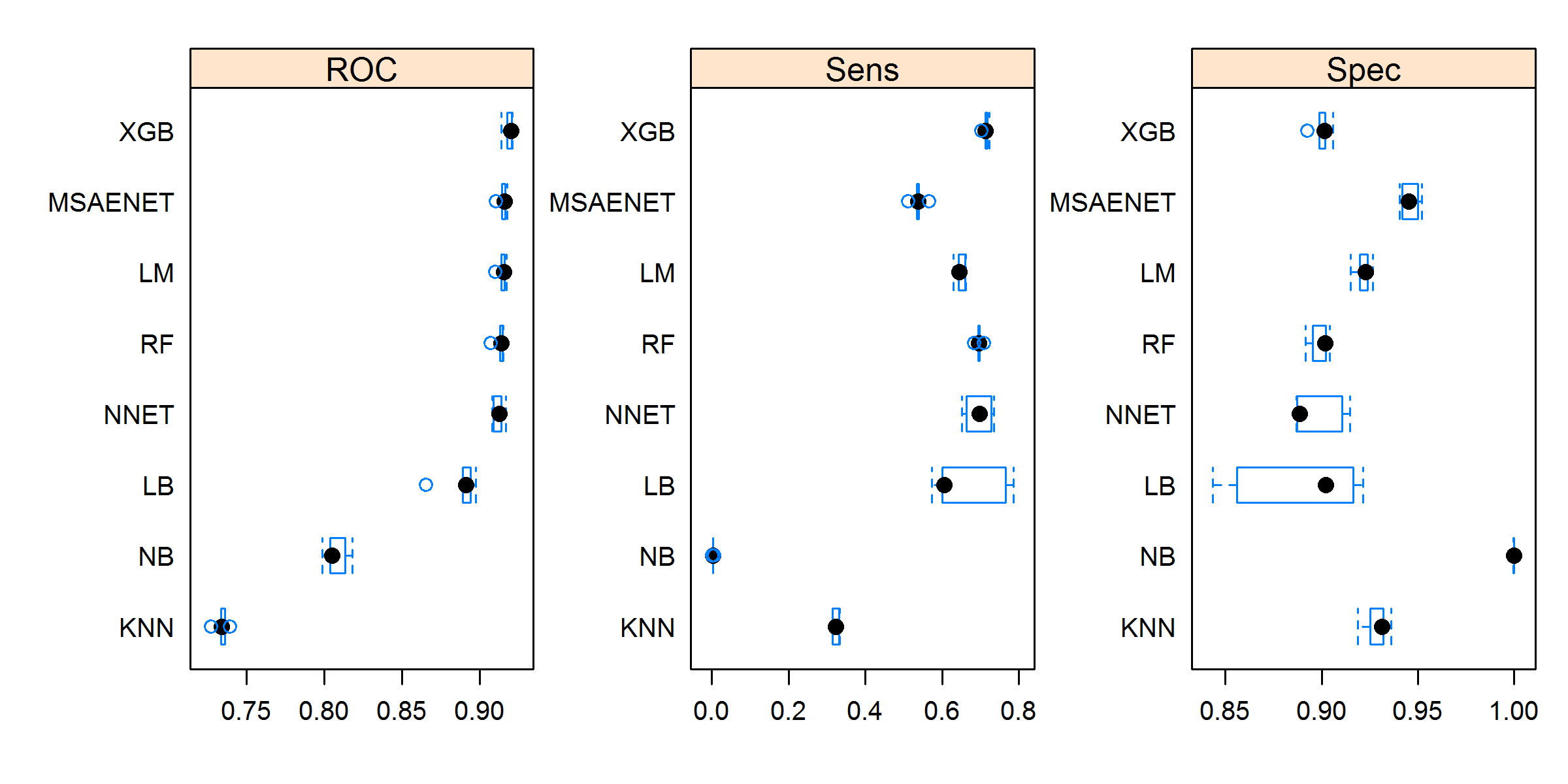

Supplement: Supplementary file 2 — Univariate increase of VAS MID to 23, hip replacement results (AUROC, Sensitivity, Specificity), no filtering. (TIFF 8437 kb) [file 12911_2018_731_MOESM2_ESM.tiff]

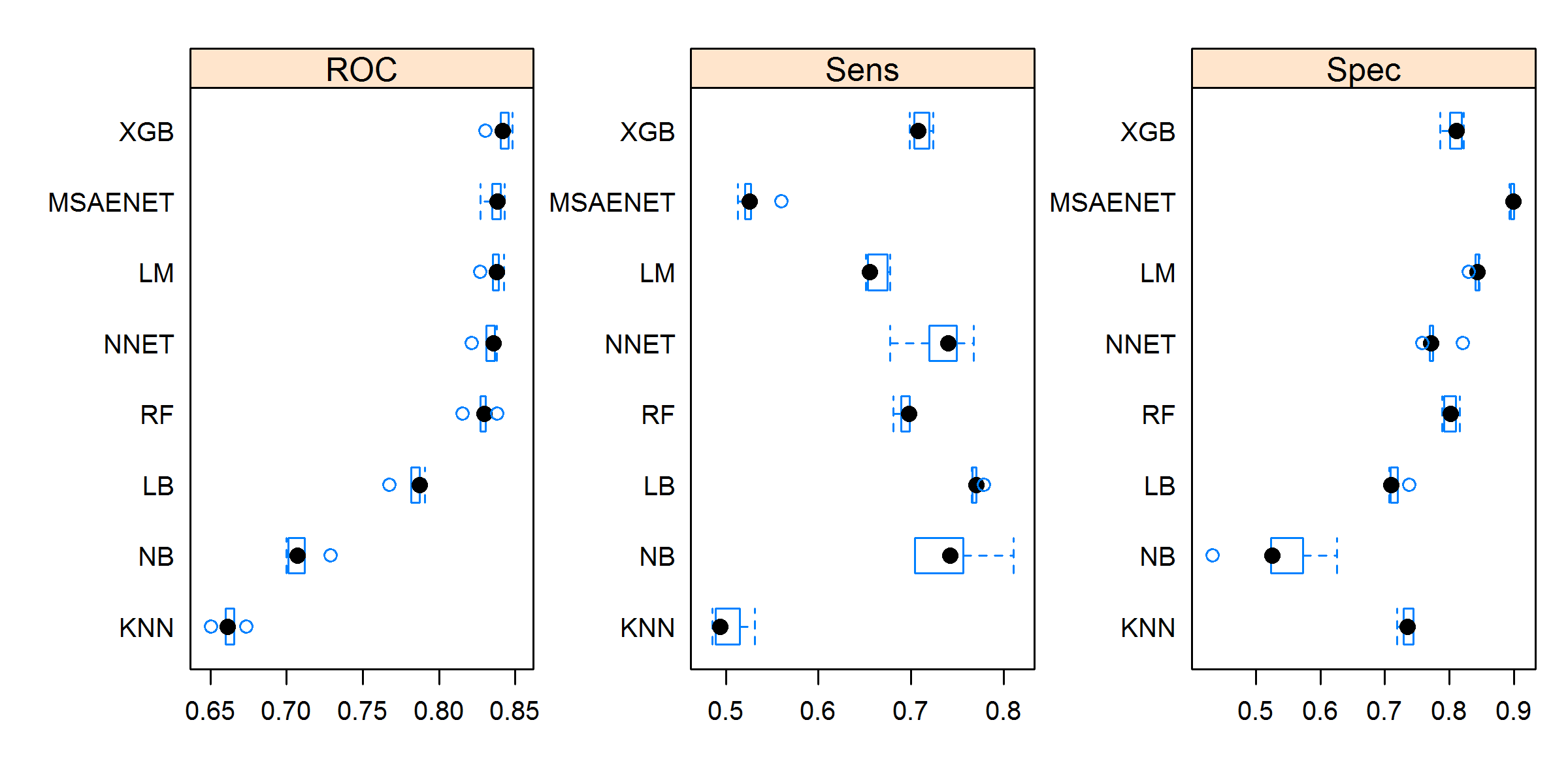

Supplement: Supplementary file 3 — Univariate increase of VAS MID to 23, hip replacement results (AUROC, Sensitivity, Specificity), filtering impossible improvement (remaining n = 19,716). (TIFF 8437 kb) [file 12911_2018_731_MOESM3_ESM.tiff]

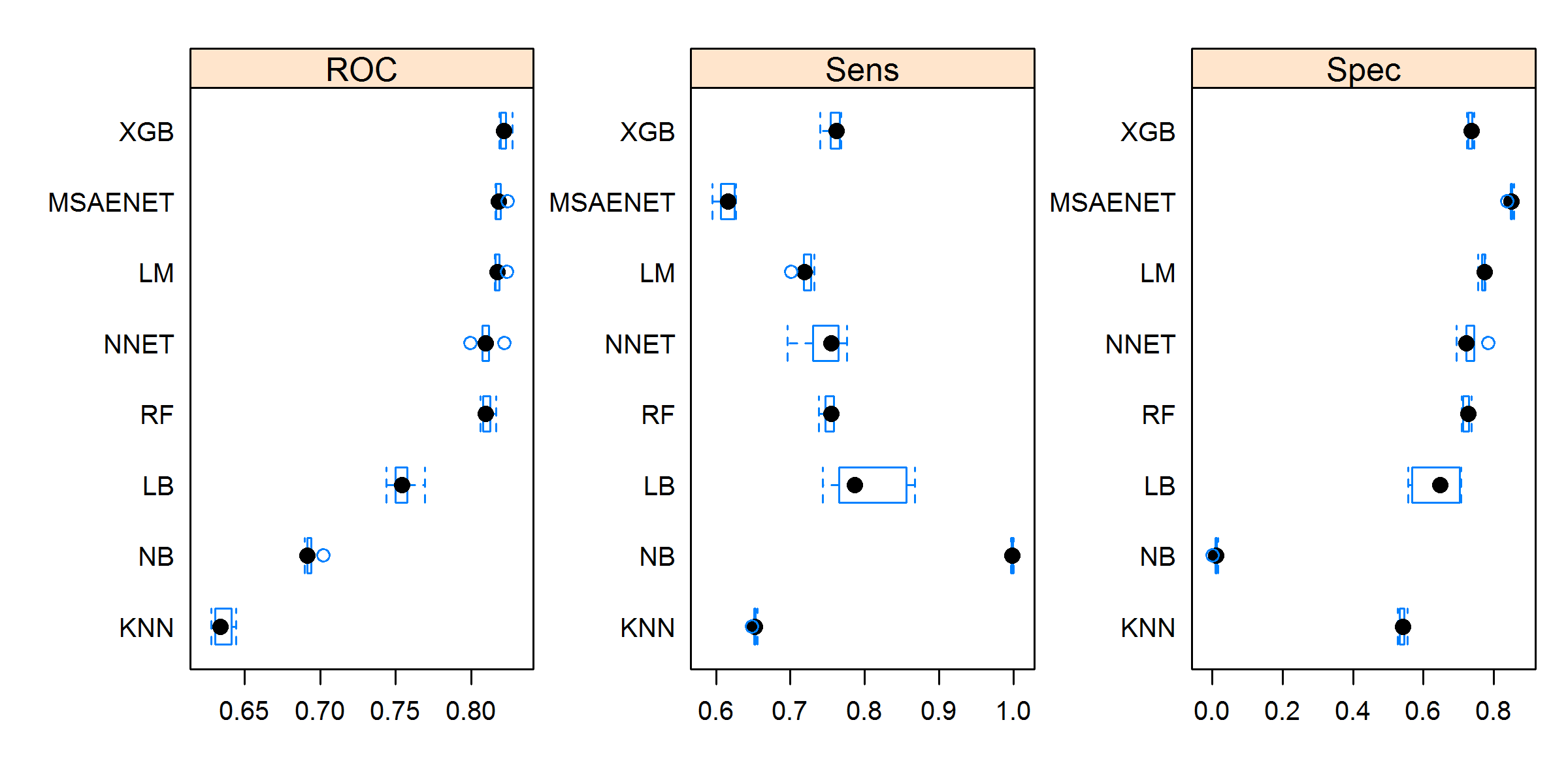

Supplement: Supplementary file 4 — Univariate increase of VAS MID to 11, hip replacement results (AUROC, Sensitivity, Specificity), filtering impossible improvement (remaining n = 25,606). (TIFF 8437 kb) [file 12911_2018_731_MOESM4_ESM.tiff]
